# Using a Clustering Method to Detect Spatial Events in a Smartphone-Based Crowd-Sourced Database for Environmental Noise Assessment

**DOI:** 10.3390/s22228832

**Published:** 2022-11-15

**Authors:** Ayoub Boumchich, Judicaël Picaut, Erwan Bocher

**Affiliations:** 1 UMRAE, CEREMA, Univ Gustave Eiffel, F-44344 Bouguenais, France; 2 Lab-STICC CNRS UMR 6285, IUT de Vannes, F-56017 Vannes, France

**Keywords:** environmental noise, noise mapping, smartphone application, spatial clustering, DBSCAN

## Abstract

Noise has become a very notable source of pollution with major impacts on health, especially in urban areas. To reduce these impacts, proper evaluation of noise is very important, for example by using noise mapping tools. The Noise-Planet project seeks to develop such tools in an open science platform, with a key open-source smartphone tool “NoiseCapture” that allows users to measure and share the noise environment as an alternative to classical methods, such as simulation tools and noise observatories, which have limitations. As an alternative solution, smartphones can be used to create a low-cost network of sensors to collect the necessary data to generate a noise map. Nevertheless, this data may suffer from problems, such as a lack of calibration or a bad location, which lowers its quality. Therefore, quality control is very crucial to enhance the data analysis and the relevance of the noise maps. Most quality control methods require a reference database to train the models. In the context of NC, this reference data can be produced during specifically organized events (NC party), during which contributors are specifically trained to collect measurements. Nevertheless, these data are not sufficient in number to create a big enough reference database, and it is still necessary to complete them. Other communities around the world use NC, and one may want to integrate the data they collected into the learning database. In order to achieve this, one must detect these data within the mass of available data. As these events are generally characterized by a higher density of measurements in space and time, in this paper we propose to apply a classical clustering method, called DBSCAN, to identify them in the NC database. We first tested this method on the existing NC party, then applied it on a global scale. Depending on the DBSCAN parameters, many clusters are thus detected, with different typologies.

## 1. Introduction

### 1.1. Noise Mapping Using Data Collected with Smartphones

Noise has become a very notable source of pollution with major impacts on health, especially in urban areas [1]. The public authorities are trying to solve this essential societal and health issue by setting up new regulations. In Europe, for example, the directive 2002/49/EC seeks to evaluate noise annoyance, to propose actions to reduce this annoyance, and to communicate with citizens about their noise exposure. In this regulatory context, the key tool for decision makers is the use of strategic noise maps.

Instead of using noise prediction software, with its inherent limits, an alternative method may be to use more affordable sensor networks, allowing us to densify the observation points [2] and, in particular, to consider the participation of citizens as data collectors in a crowd-sourcing approach using smartphones as sensors (i.e., a measuring instruments). This idea of using smartphones as acoustic sensors and citizens as contributors emerged at the end of the 2000s with the increasing capabilities of smartphones to perform environmental acoustic measurements [3]. It was followed by several works that have given rise to specific noise and soundscape crowd-sourcing-type applications and platforms (e.g., Ear-Phone, NoiseSPY, NoiseTube applications [4,5,6]) and, particularly, the NoiseCapture (NC) approach [7,8], a part of the Noise-Planet project [9].

**Figure 1:**
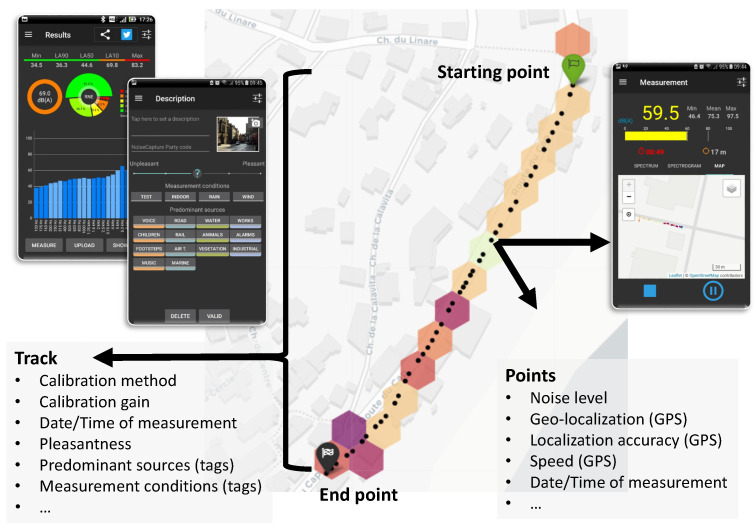
Representation of a NoiseCapture (NC) measurement. After manually activating the measurement from a starting point, the user can move along a path of their choice or stay at the same location. An acoustic measurement takes place every second, which allows us to calculate noise indicators, such as 1 s equivalent sound level and spectrum. Other information, such as the date and time of the measurements, the GPS location of the measurement point, its accuracy, and its speed are recorded at the same time. At the end of a track, the user stops the measurement and is invited to provide additional information about the presence of specific sound sources (tags) during the measurement, the measurement conditions, his own perception of the quality of the sound environment (i.e., the pleasantness), and other additional information. If the user has given his consent, the anonymous data are transferred to a remote server, controlled and then integrated into an open database, which is available for free. In addition, the raw data from the entire community are aggregated into a hexagonal based noise map and displayed on a public web page [10], as shown in this figure. Graphical contents of this figure are part of the Noise-Planet project website [11], licensed under CC BY-NC-SA 4.0.

The NC approach consists of measuring noise and additional information along a path and then sharing data with the community (Figure 1). The noise data acquired by volunteers (i.e., the “user” or the “contributor”) from all over the world are then stored in a community database. This database contains the measurement path (a “track” in the NC vocabulary, which is made of “measuring points”), standardized noise indicators, a description of the user perception of noise sources (using “Tags”) and of the soundscape quality (using a pleasantness scale), and other useful information, such as the date and time of the measurements, the GPS localization and accuracy, and the speed of the user during measurement…

This data can be relevant for later evaluation of the ambient noise through specific processing, for example, to generate noise maps that could be useful for communities to implement action plans in order to reduce the noise exposure of citizens or to protect quiet environments. In this way, the use of NC maps would be consistent with the European directive 2002/49/EC except that they simultaneously integrate the multiplicity of noise sources encountered in the environment. In comparison with conventional noise maps, for example, focusing on traffic noise only, those produced with NC data could be closer to reality.

Since the launch of the application on 29 August 2017, a large amount of data has been made available, offering the possibility to analyze the data over a large spatial area and a long period of time. This database is distributed under the Open Data License [9] and updated every day at 3:30 am (local time in Paris, France). At this date, the database represents the equivalent of more than 1103 days of 1 s measurements (more than 382,000 tracks and 93 million measurement points) spread over more than 200 countries and collected by over 92,000 different contributors.

### 1.2. Data Quality Control and the Need for a Reference Database

This amount of data, however, needs to be put into perspective, firstly with regard to the surface of the planet (150 million km^2^ for the terrestrial surface) and the time elapsed from the launch of the application (1737 days by 31 May 2022); finally, the density of points in space and over time remains globally very low (less than 0.4 measurement points per 1000 km^2^ by day) except in urban areas where the density of measurement may be more important. In addition, the quality of the collected data may sometimes be questioned. A recent analysis [8] showed that data may suffer from several problems due to the technical performance of the smartphone, respect for a relevant measurement protocol, the lack of smartphone calibration, the misuse of the application, and insufficient GPS accuracy…

The evaluation of the quality of the collected data is therefore essential to characterize the relevance of the noise maps and the analysis of the sound environment that will be produced. For example, it is important to identify wrong or incomplete data, as well as anomalies in the NC database…which is more generally called data Quality Control (QC). For this purpose, among all the possible approaches, those based on machine learning techniques have been widely used in many fields of interest [12], in particular, when considering crowd-sourced data [13,14,15,16]. Three main methodologies can thus be used in machine learning: the supervised, the semi-supervised, and the unsupervised ones, depending on whether they use a large amount of labelled data, a small amount, or none, respectively.

In the NC context, the supervised and semi-supervised machine learning methods are well-suited because of the existence of labelled data characterized by a maximum level of confidence. Indeed, in the NC approach, such data are provided during NC parties, i.e., an event that is organized by acousticians or other experts in a limited space and in a relatively short time to simultaneously collect a large number of contributions. In this case, the contributors are coached, the measurement protocols are correctly applied, and the smartphones are most of time calibrated; thus, the data collected during such an event are generally of better quality and may be considered as reference data. However, the performance of (semi-)supervised methods requires a sufficient amount of data, which is undoubtedly not the case on the basis of data produced only by the NC parties.

If most of the time NC parties are organized by close collaborators of the NC project, the recent scientific literature shows that similar events are also organized for research purposes by people who are not directly related to the NC project. More globally, such events can also be organized by communities, citizens associations, or schools during awareness-raising events on noise issues. The data collected during these events can also be considered as labelled data and should therefore be identified in the NC database.

### 1.3. Objective of the Paper

The objective of the present paper is therefore to propose a method to identify in the NC database events similar to the NC party, i.e., a set of data collected by multiple users, produced over limited spatial extent and temporal period, and most of the time with a higher spatial and temporal density of measurement points. More explicitly, regrouping data with similar spatial and temporal characteristics is known as spatial clustering and temporal clustering [17]. Note that in the following, the paper focuses only on spatial clustering, with the temporal parameters being considered as a filter for the data (this will be presented later in the document).

Many clustering methods have been proposed in the literature. Among them, the DBSCAN approach seems well-suited to our problem and is considered in the present paper. Thus, the originality of the proposed work lies mainly in the use and validation of this method for the needs of our problem and not in the development of a specific clustering method.

Some generalities on spatial clustering methods are presented in Section 2. Then, in Section 3, the DBSCAN method is detailed and tested on known NC parties in order to evaluate its performance and to identify the most appropriate processing parameters. Finally, the DBSCAN method is applied in Section 4, firstly to a few countries in order to identify the typology of the obtained clusters and secondly to the entire NC database. Lastly, Section 5 concludes this study, showing the interest of clustering methods to identify data that could be integrated into a reference database.

## 2. Spatial Clustering Related Work

Clustering is a useful method in data science [18,19], allowing us to identify similar groups of data in a data set (i.e., objects in each group are comparatively more similar to objects in other groups), which are called *cluster*. It should be noted that the term “close” is sometimes used instead of “similar”. For example, let us consider two houses (“A” and “B”) and one studio (“C”), where houses “A” and studio “C” are in the same neighborhood and “B” in a different city; if the clusters were formed to regroup housing with similar characteristics for sale, then houses “A” and “B” can be clustered together, but if we are talking about spatial clustering, then house “A” and studio “C“ could be regrouped together because they are close to each other. Therefore, it is important to pay attention to the characteristics that are considered when looking for similarity or closeness.

It must be noted that classification is another technique that has certain similarities with clustering and which could have been envisaged for the present study. Classification refers to the act of categorizing or predicting the class of any given data. Classification is supervised and demands a training data set with class labels, while clustering is non-supervised, aiming to find underlying unknown groups or clusters). In the present study, the lack of criteria, such as spatial/temporal limit, number of users, and number of tracks/points to define classes, made the clustering approach more relevant than classification.

Clustering has a large number of applications stretching out across various domains, such as recommendation engines [20], market segmentation [21], social network analysis [22], search result grouping [23], and anomaly detection [24]. Clustering analysis can also be applied to the environmental measurement data (e.g., temperature [25,26] and humidity [25,27]). Clustering is also widely used in geospatial analysis [28].

In the environmental acoustic field, clustering has already been applied to analyze the results of the ”Think About Sound“ smartphone application, using the experience sampling methods (ESM) [29]. Clustering was also used to group similar urban soundscapes using the fuzzy ants rule and K-means method [30,31] or to monitor road traffic noise by using temporal clustering methods [32,33]. Nevertheless, the last clustering methods were applied on the basis of the information contained in the collected data, and, to our knowledge, no methodology has been applied to spatially clustering the data as we plan to do with the NC data. The data collected by NC can be analysed by path (i.e., track) or by point. The applicable spatial clustering methodologies can therefore cover *Points Spatial Clustering* or *Line/Trajectory Spatial Clustering*. In the following, we will focus on *Points Spatial Clustering*.

*Points Spatial Clustering* methods that may be applicable to the present study can follow several approaches that are inspired by generic clustering methods [34,35]:

- *Partition Clustering* allows grouping data in *K* non-overlapping sub-groups (i.e., *K* clusters), one datum being in only one subgroup. One can consider several methodologies, for example, the K-means, K-medians, or K-medoids methods, depending on the choice of the cluster center, at the average point, the median point, or the point in the data set closest to the median point, respectively.
- *Hierarchical Clustering*, using the Agglomerative or the Divisive Hierarchical Clustering methods, tries to build a hierarchy of clusters using a bottom-up approach (each datum starts in its own cluster, then the two closest clusters according to a chosen distance are merged until all clusters are merged, creating a tree that one has to cut according to the relevant number of clusters) or a top-down approach (at the opposite of the bottom-up approach, all data are initially in the same cluster and, then, the cluster is split according to the hierarchy level), respectively.
- *Fuzzy Clustering* is another form of data clustering in which each datum can be included into several clusters. As a possible approach, the Fuzzy C-means method, which is the most widely used, is quite similar to the K-means clustering method.
- *Density-Based Clustering* methods propose to group data by considering the density of data. Then, each cluster is built by considering regions with a high density of data.
- Lastly, the *Model-Based Clustering* method groups data by considering that they are generated by probability distributions and that each cluster represents one given distribution.

In the framework of the NC data, it is clear that the fuzzy clustering method cannot be used in the present study since each measurement point can be associated with only one event. Considering the model-based clustering methods, since the data are not associated, a priori, with any distribution model, the method seems inapplicable. The partition clustering method, which imposes fixing the number of clusters to identify in advance, is obviously also inapplicable since it is impossible to know the number of NC events that will be found. Lastly, the use of a hierarchical clustering method is very empirical since it requires the retention of a number of clusters that should be coherent with the data, but with, a priori, the possibility of omitting clusters, in particular if the amount of data is very large. Moreover, the processing time can become considerable because of a quadratic increase of the complexity in $o(n^{2}\log n)$ (*n* being the number of data), compared, for example, with an analysis using the K-means method that is characterized by a linear increase in o(n). Finally, the density-based clustering approach seems an interesting way, since one can consider that an NC event generates an increase in the density of measuring points locally, over a short period of time. Among the possible approaches, the density-based spatial clustering of applications with noise approach (DBSCAN) method is the most commonly used [36]. It must be noted that the term “noise” that is employed in the name of the method does not refer to the environmental “noise”, but to “noise” in the data, meaning that some points may not be part of a given cluster.

Considering automatic learning, such as clustering, most techniques will either fall into (1) supervised, (2) semi-supervised, or (3) unsupervised learning. Supervised learning provides the model with both the input and the output of the data (also called labelled data), while unsupervised learning provides only the input of the data. On the other hand, semi-supervised learning provides the model with a small amount of data that contains both the input and the output and a large amount of data that contains only the input. In the NC context, supervised learning can be hard to use due to the lack of labeled data (i.e., NC party data). So either unsupervised learning or semi-supervised could be performed, but due to the lack of similarities in NC party events, it is feared that a direct semi-supervised approach will not work. Therefore, an indirect semi-supervised approach has been considered in the present work. This approach consists firstly of using the labelled data (i.e., NC party data) to perform supervised learning in order to find the optimal parameters of the DBSCAN method, and secondly, of performing an unsupervised learning task, using DBSCAN with these parameters to detect events that are similar to NC parties.

## 3. Spatial Clustering of the NoiseCapture Data with the DBSCAN Method

### 3.1. Implementation of the DBSCAN Method

**Figure 2:**
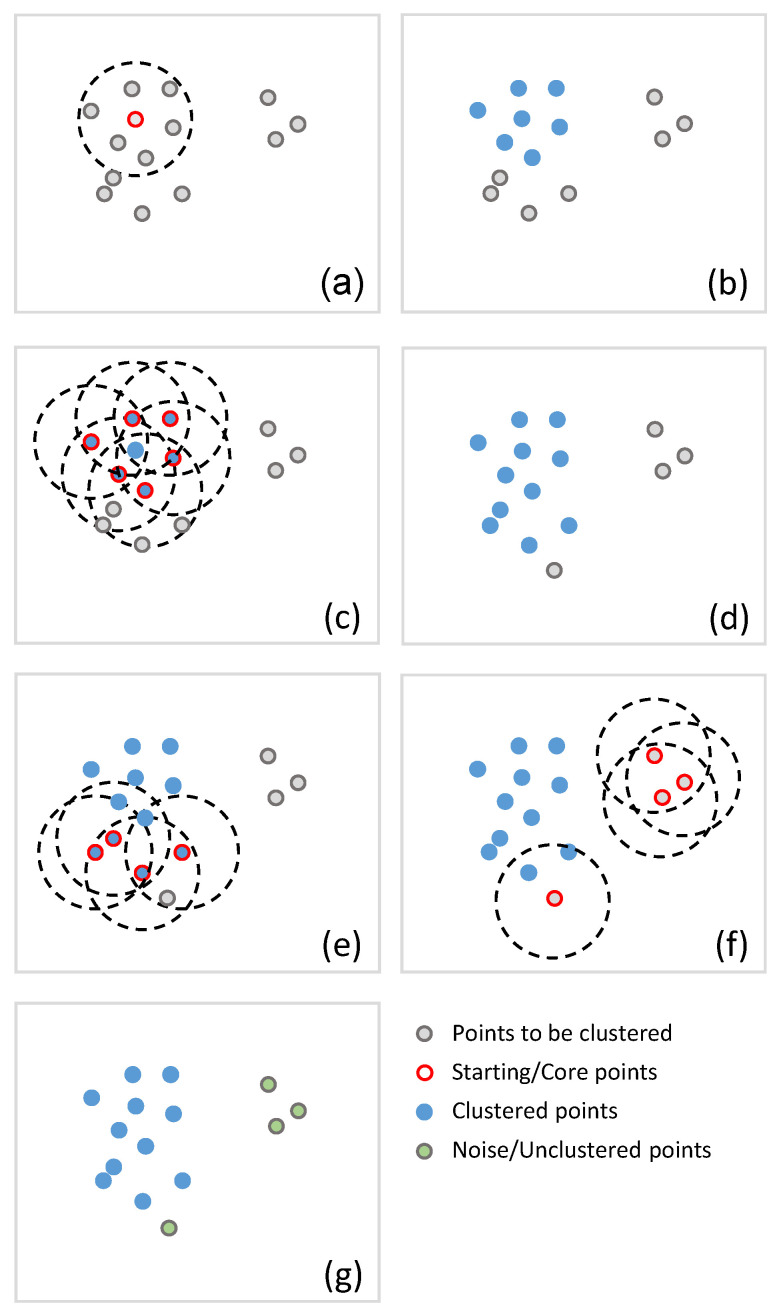
Graphical representation of the DBSCAN approach, with MinPts=4 as an example: (**a**) a starting point is first selected in a set of points to be clustered; if the number of points in a circle with a radius of Eps centered on the starting point is greater or equal to MinPts, all of the points are included in the same cluster: in this example, there are 6 points in the circle, thus the condition is validated; (**b**) a first cluster is created with the staring point and the 6 new members; (**c**) each new member of the cluster becomes a starting point; the condition presented in (**b**) is applied again: in this example, only one starting point respects the conditions, then (**d**) 4 points are included in the cluster; (**e**) the condition is applied again on the new members; in this example, the condition of a minimum of points in the circle is not verified: the process stops and a cluster is then complete; (**f**) a new starting point is selected outside the existing clusters and the process is repeated until all points have been proceeded; (**g**) in this example, 11 points have been grouped together in one cluster and 4 points are not clustered.

The principle of the method is trivial and based on very pragmatic considerations (i.e., a cluster represents a set of points very close to each other), without any theoretical basis. In the present study, the approach is reproduced as it was proposed by its authors [36]. It consists of searching a minimal set of points (MinPts) around a given point (in a circle of radius Eps) to start forming a cluster. This cluster evolves progressively by searching for new points to integrate from each of its points that are already members of the cluster. The method is thus defined by these two parameters Eps and MinPts only (Figure 2).

The method first randomly selects a point in the database. Then, it selects all the points within a circle of radius *Eps*. If the number of those points is greater than or equal to *MinPts*, then they are integrated into this cluster. At the next step, each integrated point becomes the center of a circle of radius *Eps* of a new iteration. When the number of points within one of these circles is less than *MinPts*, then the procedure stops for the corresponding center. When the search becomes unsuccessful for all the current points in the cluster, the cluster stops growing. The procedure then starts again by randomly selecting a new point in the database outside of any cluster already formed as the new center of a possible cluster. When all the points in the database have been processed, the procedure stops and eventually leads to the creation of a finite number of clusters, or none. As mentioned before, all the points that are not part of a cluster are considered “noise”.

In agreement with the NC database model that is used in the present study (cf. Section 3.2), the DBSCAN method has been implemented using the ST_ClusterDBSCAN() function available in PostGIS, a spatial database extender for the PostgreSQL object–relational database [37]. This function is a 2D implementation of the DBSCAN algorithm (the spatial clustering of the NC database is carried out on the 2D coordinates without the elevation). This function takes three inputs, geom as a geometry data type, Eps as a float data type, and MinPts as an integer data type, and returns one identifier per found cluster, as an output. The results are saved in a table called NoiseCapture_cluster, which contains the measurement point identification pk_point of coordinates the_geom and cluster identification c_id. Another variable, pk_party, is also added to verify if the corresponding point is already part of a known NC party event and, if so, the corresponding NC party identification. Finally, the data and the clusters are plotted in GIS software. Note that all the tools used in this study are open source.

### 3.2. NoiseCapture Database

The NC measurement locations are stored in WGS 84 (World Geodetic System 1984) format [38], which is a horizontal component of a 3D system used, for example, by the GPS satellite navigation system. In other words, the type of the coordinates is geographic. This encoding uses ”degree“ as a measure distance, while the ST_ClusterDBSCAN() function only takes coordinates as geometry data type. Therefore, a transformation to the metric projection EPSG:3857 (Pseudo-Mercator) [39] is required before applying the algorithm. This projection can only be used for data between 85.06° S and 85.06° N, which is in agreement with the NC database (NC data are bounded between 74.49° S and 78.73° N). The transformation was performed using the PostGIS function ST_Transform [40].

The DBSCAN algorithm was used on a 3-year extraction of the NC database, from 29 August 2017 to 28 August 2020, starting from the official release version ”28“ of the NC smartphone application [41]. Any measurement point in the NC database without geo-localization (may be a full track or only a part of a track) was removed before processing by the DBSCAN method. Using this NC database extraction, all the data can be organized into a relational database, which can be manipulated using GIS tools.

In order to find the optimal parameters for DBSCAN, we use official NC party events as reference data. The objective of the first part of the study is to find the parameters of the method that allow us to find a maximum number of NC parties in the form of clusters, or at least, the best detection rate. During the corresponding 3-year period, 27 NC parties were planned [8] (Table 1), but one of them (i.e., NC party number 31 with code name CICAM) was not performed. The training data set has 26 NC parties, spread over 3 years. These NC events took place in France (18 NC parties, one including an event also shared with Germany), in Spain (5 NC parties), in Italy (2 NC parties), and in The Netherlands (1 NC party). During these events, 3142 tracks were collected, for a total of 391,575 measurement points. Around 377,206 (96.33%) points were geo-located, and 320,891 (81.95%) had accuracy less than or equal to 15 m; 3012 (95.86%) tracks were completely geo-located (i.e., all the points of a track are geo-located), and 2641 (84.05%) tracks had accuracy less or equal to 15 m for all corresponding points.

NC party events may be defined by the organisers with two parameters: the filter_area and filter_time, which stand for the spatial and temporal limits that were decided for each NC party. Four NC parties (15.38%) did not have a spatial limit, while only two (7.69%) did not have a temporal limit. Statistics showed that ten (38.64%) of NC parties had points outside their limit area and that three (11.54%) NC parties had measurements outside the time limit; in this last case, measurements were all dated in the past (in 1994, 1999, and 2000), expressing a problem with time synchronization of some smartphones. In terms of measuring points, it means that 87.28% points (85.13% tracks) were collected within the area limit and 91.06% points (93.03% tracks) within the time limit. When both the area and time filters were used, 86.28% of the points (84.3% of the tracks) of NC parties were collected within the time and space constraints. The duration is specific to each NC party and is between 2 and 10 days, with an average of around 5 days (less than a week).

The number of events (26) and their amount of tracks/points represent only 1.2% of the NC database and 0.6% of the points. It may not be enough to assess the optimal parameters for DBSCAN.

### 3.3. Filtering Variables

In addition to the two parameters Eps and MinPts, one can also consider supplementary parameters in the DBSCAN methodology in order to reduce the amount of data to consider by pre-filtering the NC database. The corresponding filtering variables may have an impact on the computational duration of the process or can contribute to increasing the quality of the clustering results:

**Time** **window** The temporal dimension is not considered in the classical DBSCAN method, which implies that in the context of NC all data are considered simultaneously, just spatially, without any consideration of date. It can then be difficult to identify NC events of relatively short duration if the corresponding measurement points are ”drowned“ in the mass of data that can be collected progressively (i.e., out of a particular event), to the same spatial extent but over a long time. As mentioned above, the past NC parties have durations of a few days. Thus, it seems interesting to test the DBSCAN methodology, filtering the NC database to focus only on data collected over a ”day“, a ”week“ or a ”month“.
**GPS** **accuracy** The accuracy of the DBSCAN method is necessarily based on the accuracy of the location of the measurement points; if the points are poorly located, then their membership in a cluster may be questioned. In the NC application, the localization of the measurement is based on the GPS system of the smartphone. In some cases, the measurement points may not be located at all; in this case, as mentioned before, the corresponding measurement points were removed from the database. For the remaining points, the associated location uncertainty can reach several tens of meters. The variable related to the accuracy of localization can also be an important element in the quality of the clusters obtained by the DBSCAN method. The method will therefore also be tested by filtering the data on the GPS accuracy values.
**Zone** **of** **study** The search for clusters depends on the number of points in the database and thus, in particular, on the size of the study area. The larger the study area is, the longer the processing time will be. Reducing the study area to territories in which NC events are potentially expected will reduce the computational time.

### 3.4. Validation of DBSCAN

#### 3.4.1. Methodology

As mentioned before, the objective of the preliminary study is to find the parameters of the DBSCAN method that allow for the best detection rate of NC parties in the form of clusters. Thus, a series of experiments were performed on the NC data by varying the values of the two DBSCAN parameters Eps and MinPts and for different filtering conditions:

- Eps was started from 50 m as the initial distance and then gradually increased (by 50 m between 50 m and 500 m, then by 100 m from 500 m to 2000 m and finally by 500 m from 2000 m forward) until a maximum of NC party events were detected as clusters.
- MinPts was started from 20 points as the initial value and then gradually increased (firstly 50, then 100, and finally by 100 until the maximum number of points for each respective NC party event) until a maximum of NC party events were detected as clusters.
- Time window: due to the typology of current NC parties, the clusters analysis was performed by filtering the NC database by day, by week, and by month, on a total duration that includes all the NC points (i.e., if an NC party took place over a period between 2 months, the 2 months concerned were fully considered).
- GPS accuracy: the DBSCAN process was performed with two settings for the GPS accuracy: “Off”, meaning that the GPS accuracy is not considered; “On”, meaning that measurements with a GPS accuracy strictly greater than 15 m are removed from the data.
- Zone of study: in order to reduce the computational time, the zone of study was reduced to the spatial areas that contained the current NC parties. However, the area must be large enough to avoid edge effects, especially if the points of a cluster are too close to the spatial boundaries.

The quality of the clustering analysis, i.e., the success in the detection of NC parties, was evaluated, on the one hand, qualitatively on the basis of maps by comparison between the obtained clusters and the points of the NC parties, and on the other hand, quantitatively on the basis of the detection rates of tracks and points. Since the cluster analysis was performed on the points and not on the tracks, the detection rate of the tracks must be evaluated on the basis of the percentage of the corresponding points belonging to the cluster. In the present case, an entire track was considered to be part of a cluster if at least one point of the corresponding track was part of the cluster. However, if only one point is concerned, and this point is integrated into a cluster by mistake (for example, due to a high GPS accuracy value), this may introduce a bias.

#### 3.4.2. Results

All the results have been summarised in a table, with the processing parameters and the detection rates of the NC parties, both in tracks and in points. More than 600 trials were performed. Results of the 600 trials are summarized in Table 2. Some of the results for these 600 trials are presented in Table 2.

**Figure 3:**
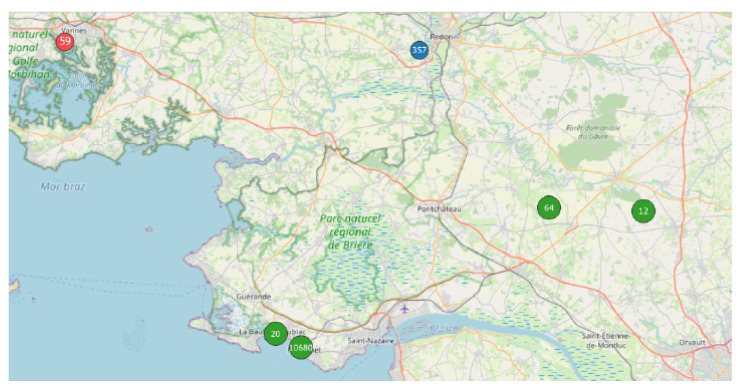
Measurement location for the NC party event Id N°1 (cf. Table 1). To simplify the representation, the points are grouped into 5 main zones. For each zone, the number of measurement points is indicated in the circle. In total, 11,192 geo-localized points have been collected during the official day of the event on 20 September 2017 (green circles), with additional points before (red circle, on 19 September 2017) and after (blue circle, on 23 September 2017) the official day. The collection points cover 2 administrative regions, “Pays de Loire” (20 + 10,680 = 10,700 points), which corresponds to the official spatial zone of the NC party, and “Britain” (59 + 357 + 64 + 12 = 492 points); represented area of approximately 44.0 km × 102.0 km.

**Figure 4:**
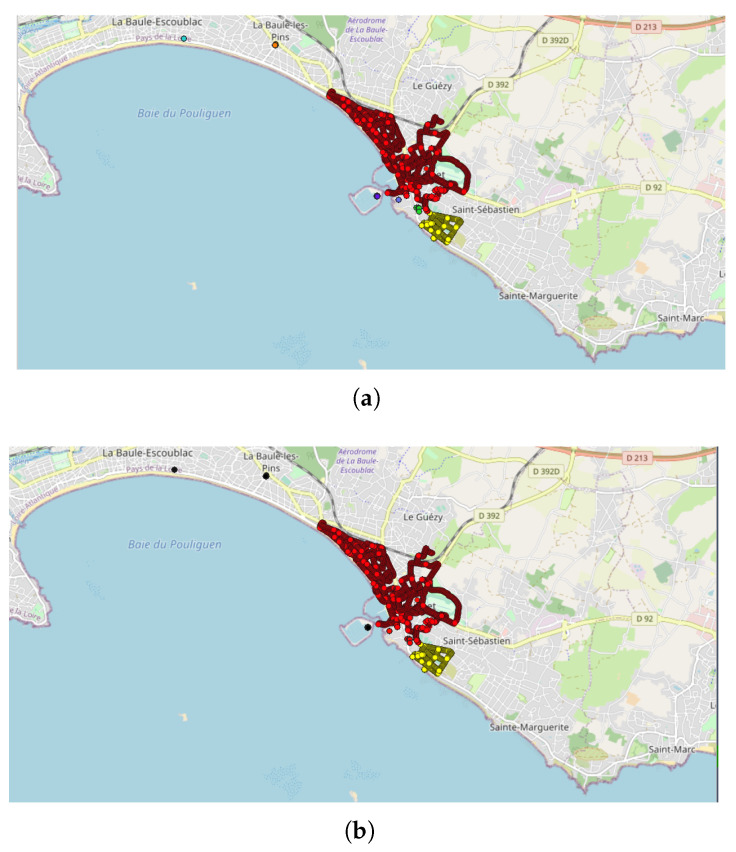

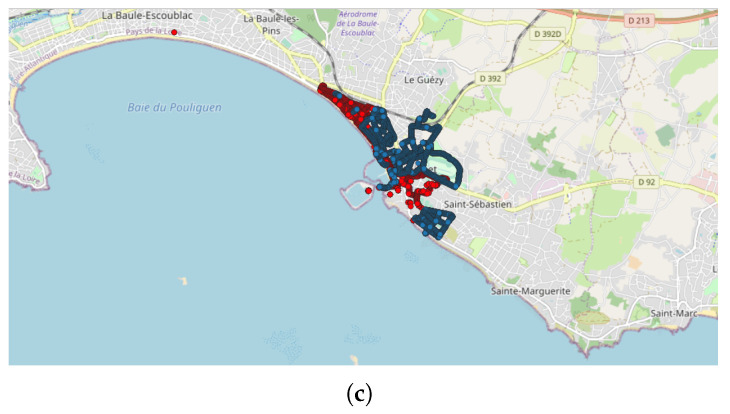
DBSCAN clustering approach, applied to the NC party Id N°1. Due to the scale of the map and because the measurement points are close together, most of the points are stacked, which makes it harder to see all of them individually. Represented area of approximately 6.3 km × 11.5 km: (**a**) representation of line #1 of Table 2 (Eps = 50 and MinPts = 20): red points represent the main cluster; the other colors represent the secondary clusters; all NC points are grouped in 7 clusters but mainly in one main cluster (red) and one secondary cluster (yellow); (**b**) representation of line #2 of Table 2 (Eps = 50 and MinPts = 100): red points represent the main cluster; yellow points represent the secondary cluster; black points represent the non-clustered data; all NC points are included in 2 clusters, the main and a secondary one; (**c**) representation of line #10 of Table 2 (Eps = 3000 and MinPts = 20); all data are located in only one cluster: red points represent all the points of the NC party Id N°1 that are part of the cluster; blue ones represent the extra data that are part of the same cluster, but not from the NC party.

For example, line #1 refers to the analysis carried out on the NC party N°1 (Figure 3), inside the spatial area ”Pays de la Loire” (PdL, i.e., an administrative zone) in France (FR), made of 10,700 points within 113 tracks, using the DBSCAN parameters Eps = 50 and MinPts = 20, after filtering the database in order to retain the data collected during 1 month only (i.e., the month in which the NC party measurements were taken). Using these parameters, 7 clusters are found (Figure 4a): 93.5% of the NC party points (10,008/10,700 points) are clustered in one main cluster, which corresponds to 91.2% of the associated tracks (103/113); the remaining 692 points (6.5%, which corresponds to 10 tracks, 8.8%) are clustered in 6 “secondary” clusters. In addition, the main clusters contain 12,212 extra points (76 extra tracks), i.e., points/tracks that are not part of the NC party. When applying the same DBSCAN parameters but changing the time window to 1 week (line #8 of Table 2), the results show that the main cluster contains the same number of tracks and points, while the number of extra data decreases slightly (12,212 extra points and 76 extra tracks). Such results may be expected when the event duration is less than a week, which is the case in this example. Lastly, when the time window is fixed to 1 day (line #10 of Table 2, which is the official day of the event), all the NC party points are clustered into only one cluster.

**Figure 5:**
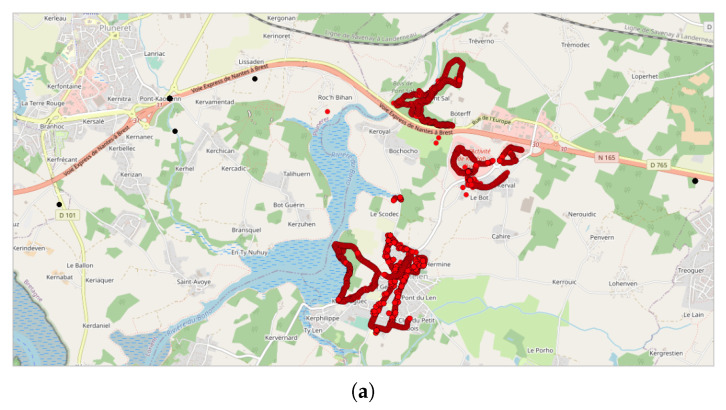

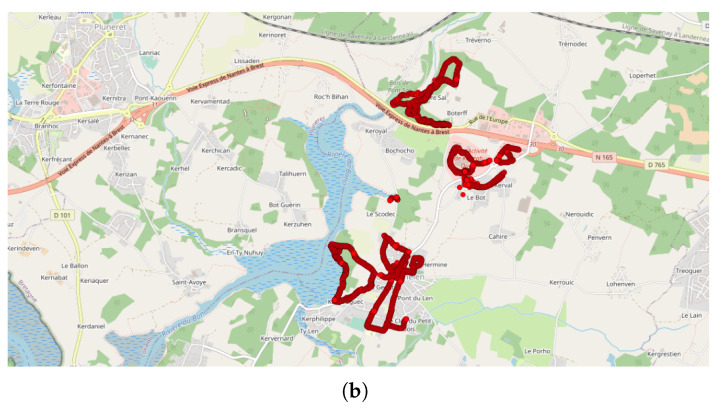
Representation of the clustering results for the NC party Id N°13 in the “Morbihan” department in France (lines 18 and 19 of Table 2). It compares the two options “Off” and “On” for the “Accuracy” filter. Only one cluster is found: red points correspond to the points of the cluster (NC party + Extra points); black points correspond to the points of the NC party that are not clustered (5 “isolated” black points: 4 on the left and 1 on the right of Figure 5a); represented area of approximately 3.4 km × 5.6 km; (**a**) “Accuracy” = “Off”; (**b**) “Accuracy” = “On”.

When the GPS accuracy filter is considered (i.e., “Acc. = On”), as in line #7 in Table 2, the number of tracks and points in the NC party decreases, since some of them are removed from the analysis; in this specific case, one can observe that 100% of the NC points/tracks are found in only one cluster, which confirms that the GPS accuracy may have an impact on the quality of the clustering. This is also illustrated in Figure 5 showing the effect of the accuracy filter for the clustering of the NC party Id N°13. In this example, several measurement points with bad accuracy are removed when the accuracy filter is enabled; all the remaining points are found in the same cluster.

Considering NC party N°1, the number of extra points is very important, the obtained cluster being about twice that of the original NC party. Looking more precisely at the time stamp and the measurement area of these extra points, one can observe that they are very consistent with the specific data of the NC party (Figure 4c). The experience of the NC party organizers shows that in some cases, the participants in the event may forget to mention the NC party code when collecting the measurements. The corresponding data are therefore not counted in the NC party but are nevertheless well associated with the event. In the present case, this hypothesis seems more than likely. An analysis of the extra points shows that 100% of the points would be well associated with the NC party. In absolute terms, this shows that the clustering method worked rather well in this case since it would have allowed the integration of unexpected but relevant data.

Lines # 1, 4, 5, and 6 of Table 2 for NC party N°1 show that increasing the Eps parameter increases the ratio of NC point/track detection (more quickly for NC tracks). This is of course an expected and obvious result, since by increasing the search radius of the points, without increasing the MinPts, it is easier to find new members for the cluster. This may suggest that the two parameters Eps and MinPts cannot be chosen independently.

This interrelation of the two parameters, Eps and MinPts, can be analyzed through a contingency-table-based analysis. The combinations of DBSCAN parameters that succeed the most in clustering 100% of the NC party tracks/points are MinPts = 20 points with Eps = 3000 m (21 NC parties are found out of the 26, i.e., 80.8%) and 2000 m (16 NC parties are found out of the 26, i.e., 61.5%). Nevertheless, these combinations worked for the NC parties in France, Italy, and The Netherlands only. Regarding the “Zone” and “Accuracy” filters, the analysis of variance (ANOVA) does not allow us to conclude on their effect on the clustering due to lack of ANOVA assumptions (i.e., homogeneity and normality). In addition, a Principal Component Analysis (PCA) was also performed, but here again, it was ineffective to describe the relationship between variables. Lastly, considering a linear regression analysis, it is observed that not only the Eps and MinPts parameters, but also the “Time window” filter, contribute explaining information regarding “Clustered data” and “Extra data”. Nevertheless, the information that is explained is rather weak; it can help when choosing optimal DBSCAN parameters and filtering variables, but they cannot be expected to be the only factors to consider. Overall, due to the small sample size (i.e., only 26 NC parties with a few points/tracks), the result is quite hard to interpret, and the statistical analysis cannot be performed under the best conditions.

Therefore, it is clear that the choice of DBSCAN parameters and filtering variables will need the experience of an “expert” to achieve the optimal clustering analysis. There are probably no optimal values, and the choice will mostly depend on the nature of the clusters to obtain. To be very selective, one should decrease the value of Eps and increase that of MinPts. On the other hand, if we simultaneously choose a value of Eps that is too high and a value of MinPts that is too low, the number of clusters may be considerable, with the risk that they are not at all representative of a specific event. Nevertheless, it is well shown that this DBSCAN approach may be useful to find clusters of interest for NC data analysis.

## 4. Application of DBSCAN on the NoiseCapture Database

### 4.1. Preliminary Results of DBSCAN in Some Countries

At this stage, one can already apply the DBSCAN method to the NC database. One of the objectives of this first application is to determine if the method is able to detect clusters and then to identify the typology of the clusters that are obtained by this approach and their possible interest.

As mentioned above, the values for Eps and MinPts parameters will condition the relevance of the detected clusters. The role of the expert is therefore important in the choice of these parameters. In the present case, the parameters are fixed to Eps = 3000 m and MinPts = 200 points, in order to limit the number of detected clusters and focus on larger ones. Indeed, the use of the previously identified set of parameters (Eps = 3000 m and MinPts = 20 points) on a large spatial area leads to a number of clusters that is too large to allow a relevant analysis.

**Figure 6:**
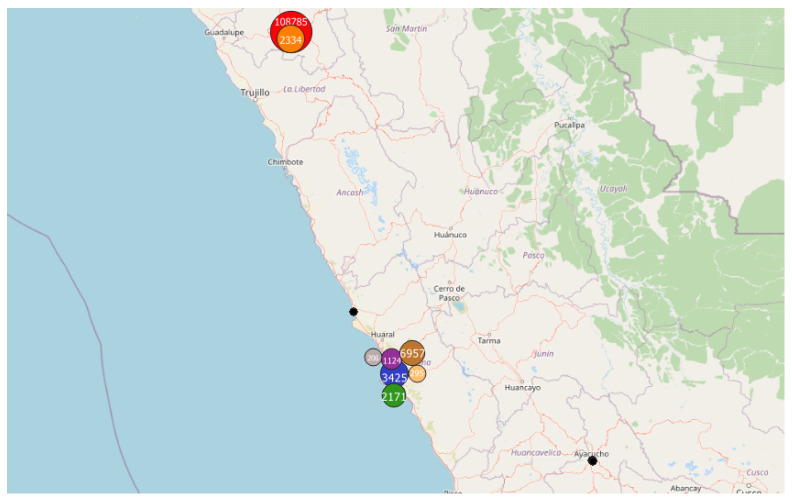
Representation of the 8 clusters found in Peru (the black points represent the non-clustered data), using the DBSCAN approach with Eps = 3000 m and MinPts = 200 points; represented area of approximately 750 km × 1200 km.

**Figure 7:**
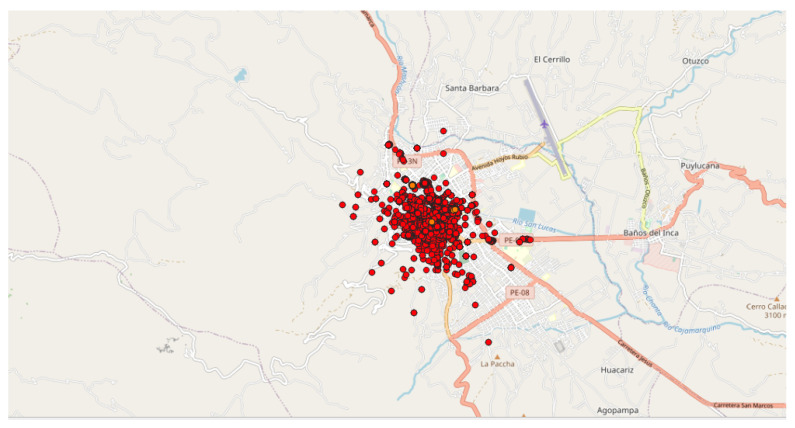
Representation of the clusters found in Cajamarca: red cluster: 10,740 tracks/108,785 points in October 2018; orange cluster: 248 tracks/2334 points in November 2018); represented area of approximately 10 km × 20 km.

**Figure 8:**
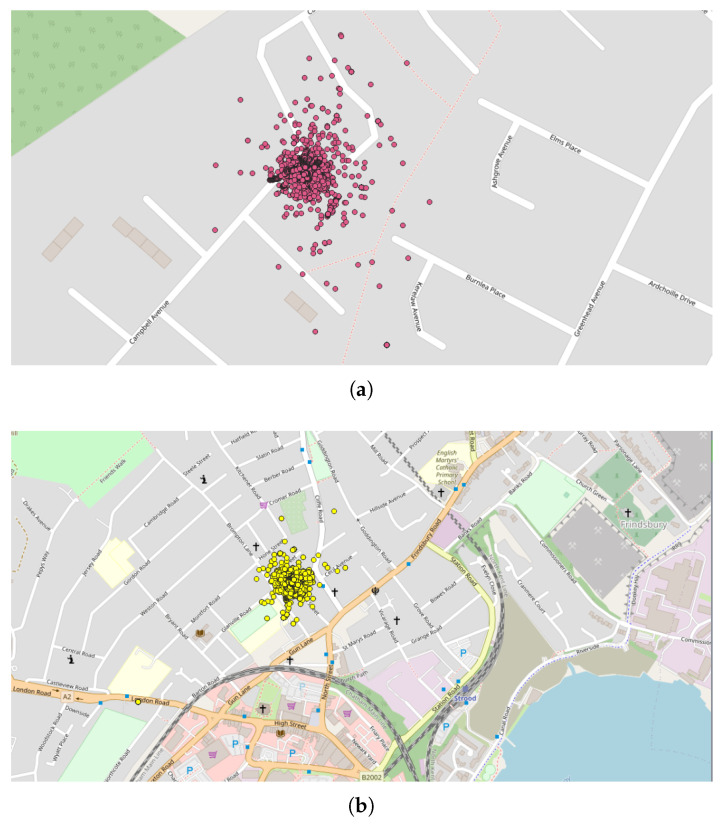
Representation of the clusters found in Stevenston (Scotland, October 2019) and Strood (England, November 2018): (**a**) Stevenston cluster (in pink); represented area of approximately 195 m × 300 m; (**b**) Strood cluster (in yellow); represented area of approximately 0.86 km × 1.70 km.

**Figure 9:**
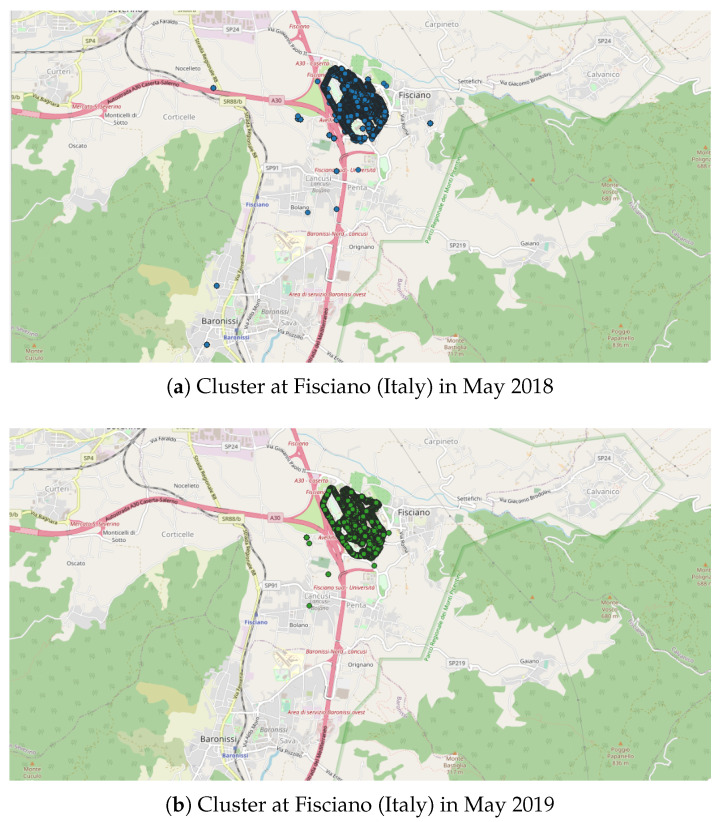
Representation of the 2 clusters found in Italy, at Fisciano. Both clusters correspond to NC party events: (**a**) NC party Id N°10 in May 2018; (**b**) NC party Id N°26 in May 2019; represented area of approximately 4.5 km × 8.9 km.

In this preliminary study, the DBSCAN method is applied to whole countries (i.e., the “spatial zone” is equal to a country), with all the geo-located data (i.e., “Accuracy” fixed to “Off”) and considering the data collected each month (i.e., the “Time window”). In this application, four countries were selected in regard to past observations [8]:

- The first application was carried out in Peru since an unusual and large amount of collected data was observed on 8–9 October 2018 in a past study [8]. Applying the DBSCAN method leads to eight clusters (Figure 6 and Table 3). Among them, one of the most important clusters effectively took place in October 2018 in the City of Cajamarca (Figure 7, green points, 10,740 tracks, 108,785 points). Another cluster was also identified in November 2018 (Figure 7, pink points, 248 tracks and 2334 points) at the same place. The tracks for the green cluster were collected by 23 contributors in 18 days, with a high concentration of measurements on a few days. Moreover, the highest number of points were collected between 09:00 and 09:59 (18.6% of points) and between 13:00 and 13:59 (17.2% of points). For the pink cluster, data were collected in 2 days by 3 users, who have also participated in collecting tracks for the green cluster. Moreover, most points were collected between 07:00 and 07:59 (90.8%). Considering the distribution of measurements over time and the total number of measurements, it is likely that these two clusters are the result of specifically coordinated events.
- The next application was realized in the United Kingdom, one of the top contributors to the NC database with 4693 tracks (4th in tracks contribution) and 2,067,182 points (3rd in points contribution). Applying the DBSCAN method leads to 440 clusters. The cluster with the highest number of points (126,533 points with 33 tracks) took place in October 2019, close to the city of Stevenston in Scotland (Figure 8). This cluster was collected by one user only during 12 days, with a few tracks per days; the highest number of the data collection was performed between 22:00 and 01:59 (25.8% points) and between 06:00 and 06:59 (8.5% points), which could suggest that an objective of the measurements was to evaluate the noise distribution during late night and early morning. The metadata show that the smartphone was calibrated for all the measurements, but the calibration value (40 dB) seems excessive in relation to what can normally be expected. The cluster with the highest number of tracks (248 tracks for 38,104 points) took place on November 2018 in the city of Strood in England. This cluster was gathered by two users, and all of the tracks were calibrated to the same value (0 dB). This cluster was collected in 21 days, with 10 to 20 tracks per day, mostly between 16:00 to 17:59 (12.1% points).
- Italy was also considered, since it is also one of the major contributors to the NC database with 2654 tracks (ranked 11th) and 364,613 points (ranked 18th) and because few NC parties have been organized. Applying the DBSCAN method gives 151 clusters, among them, the two known NC party events, which took place in Fisciano in May 2018 and May 2019 [42,43] (Figure 9).
- Lastly, the DBSCAN method was also applied to France, where most of the NC parties were carried out and potentially, some non-official events. A total of 1852 clusters were found, among them 211 during the month of September 2017, which corresponds to the first month of the application’s existence. The cluster with the most tracks and points (1358 tracks/32,0850 points by 429 contributors) is observed in Paris in September 2017. This cluster was collected during the entire month with a majority of points collected on 11, 12, and 13 of the month, with most points collected between 12:00 and 12:59, 10:00 and 10:59, and 19:00 and 19:59. Using corresponding DBSCAN parameters in the case of France, the method returns too many clusters to make a detailed and individual analysis. It is also unlikely that all these clusters are associated with events. This suggests that the number of clusters should perhaps be limited to be sure of their interest.

### 4.2. Cluster Typology

The last application shows the ability of the DBSCAN method to identify clusters of interest, for which the observed behaviors suggest that they are specifically organized events. Among the large number of clusters detected, not all can give rise to the same attention. The analysis of the clusters obtained in this first step allows us to identify the following cluster typology:

- Type A (38.0%): Clusters composed of a large number of tracks/points and with data collected by multiple users during a period of a month or less. This is the most expected cluster type, since it typically corresponds to an NC party type event.
- Type B (40.7%): Clusters with data that are collected by one or a few users express the involvement of one or a few people in the collection of a large number of measurements. It is a priori an individual behavior, which can illustrate the involvement of some people in the “crowd-sourced” spirit of the NC project. This type of cluster can be interesting, especially if the user is considered an expert.
- Type C (4.4%): Clusters composed of a lot of tracks but with a small number of points in total.
- Type D (13.5%): Clusters that are composed of only one track and contain a few points (between 200 and 500 points).
- Type E (3.4%): Clusters with a regular daily collection for more than several days.

Other behaviors were also observed, which would suggest that it is possible to combine several clusters into the same cluster:

- Clusters of data collected during the same time period (day or hour) but in different locations.
- Clusters that are close together and may be related to the same event.
- Clusters of data collected by the same users in the same location but at different times.

Based on the previous parameters for DBSCAN, it is clear that the number of detected clusters can be very important once applied to the whole NC database. Moreover, based on this typology of clusters, and depending on the final goal of the clustering, not all clusters have the same importance. Coming back to the initial objective of the present study, i.e., the constitution of a reference database, type A and B clusters are certainly the most relevant due to a much higher density of measurement points, expressing a willingness to collect data over a given spatial extent. On this basis, the higher the value of the parameter MinPts will be, the larger the number of measurement points that will be important and the more the number of concerned clusters that will decrease (as shown by Table 4). As also expected, regrouping data by month decreases the number of clusters, since a cluster associated with the same event over two weeks will be separated into two distinct clusters if the search is performed by week instead of by month.

### 4.3. Applying the DBSCAN Method to the Full NC Database

As a second application of the DBSCAN approach, the full NC database is considered (197,568 tracks, 48,901,719 points, 50,868 contributors, 195 countries), using the parameters Eps = 3000 m and MinPts = 5000 points, filtering the data weekly. On a laptop (Intel(R) Core(TM) i5-10210U CPU 64-bit Processor), the method takes about 16 h to process without any special optimization.

**Figure 10:**
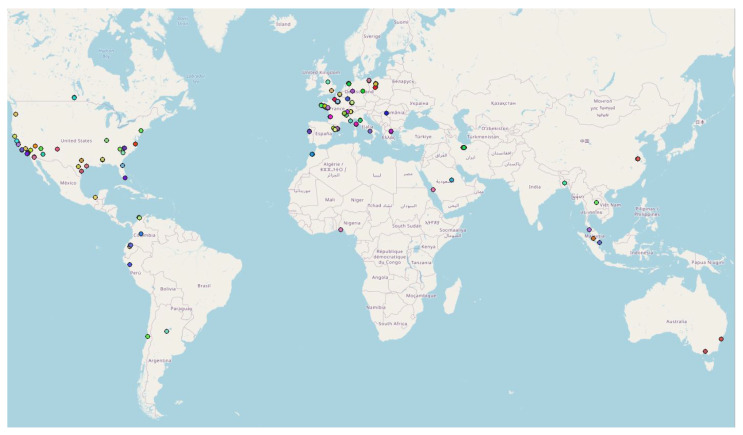
Representation of the 227 clusters with at least 3 contributors found around the globe using the DBSCAN approach (1 point is a cluster; cluster points may overlay; the color of each cluster is arbitrary).

Overall, 2046 clusters were found in 68 countries. The United States showed the most clusters (975 clusters), followed by France (297 clusters) and the United Kingdom (111 clusters), which is an expected result since these three countries are considered among the top three contributors to the NC database [8]. Among these 2046 clusters, 1567 clusters (76.59%) were collected by one contributor each (found in 40 countries, by 1155 different contributors). In addition, 252 clusters (12.32%) were collected by two contributors each, in 31 countries. This leaves 227 clusters (24,280 tracks and 4,548,638 points) with at least three users contributing to the data collection for each cluster (Figure 10): 95 clusters in France, 36 clusters in United States, and 9 clusters in Switzerland. Moreover, 19 of these clusters were NC party events.

The analysis of the literature shows that scientific studies have also been carried out on the basis of the collection of measurements using the NC application by teams without any link with the NC project team. At this stage of the study, it seems interesting to check if corresponding clusters have been found by the DBSCAN approach for these specific experiments.

**Figure 11:**
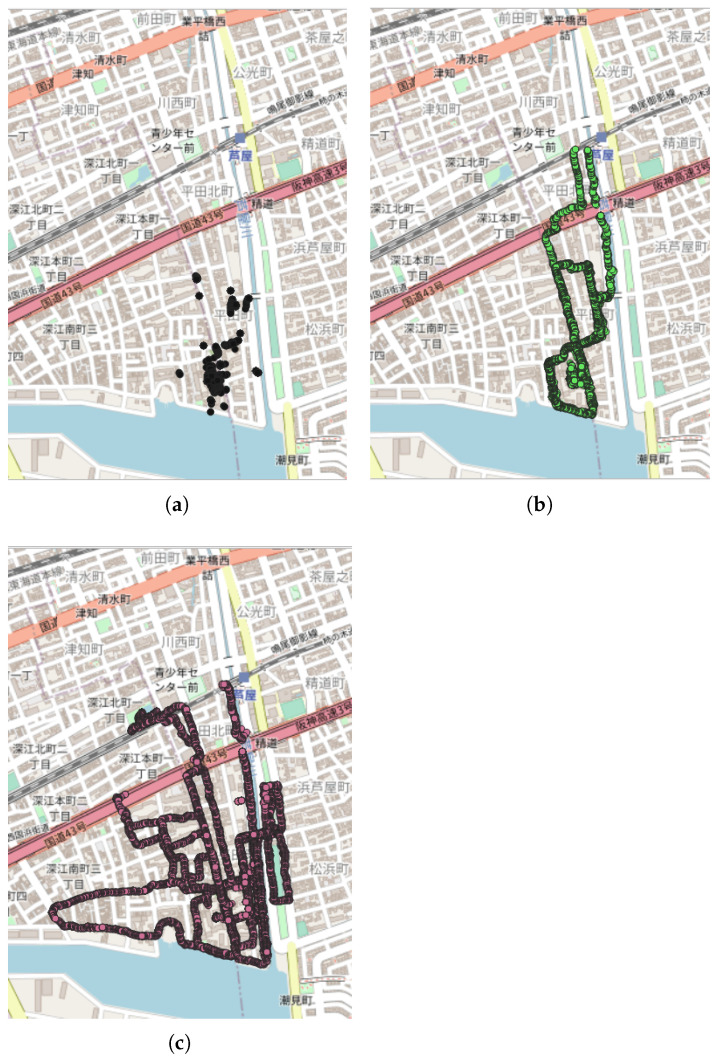
Representation of the clusters found in Kobe (Japan), in 2020; represented area of approximately 1.3 km × 1.05 km: (**a**) representation of the measurements carried out during the event [44] in May 2020 (190 tracks, 3850 points); (**b**) [representation of the measurements carried out by the same users of the event [44]. July 2020 (28 tracks, 7778 points); (**c**) representation of the measurements carried out by the same users of the event [44] in August 2020 (12 tracks, 15,928 points). All the data are extracted from the NoiseCapture database [41].

A first study, published in 2020, was carried out in Japan [44] in order to evaluate the effect of COVID-19 pandemic lockdowns in the eastern edge of the city of Kobe. An NC measurement campaign was set up over a period of one hour (10:00–11:00 am), with an average time of 30 s, at six locations in an urban area (as well as at a fixed position in front of a building), on two different days in May 2020, during and after the lockdown period. Using the clustering approach, the measurements related to the study were found as non-clustered data (Figure 11a). This is probably due to the number of measurement points (3850 points) that was a priori lower than the number of measurement points for detecting clusters (MinPts = 5000 points). Nevertheless, two clusters were detected in the same area in July 2020 (Figure 11b) and in August 2020 (Figure 11c). These measurements campaigns were carried out by the same two users that carried out the study in May 2020 [44], which may indicate additional measurements to the initial experimentation.

An NC measurement campaign was also carried out in India [45] in three urban zones corresponding to specific noise ambiances of Lucknow City: “Polytechnic chauraha”, “Hazrat ganj chauraha”, and “Haniman chauraha”. The measurements were collected at three time periods of the day (morning, afternoon, and evening), for 10 min each. However the number of measurement points was a priori not sufficient for the clustering methodology to detect this event as a cluster.

**Figure 12:**
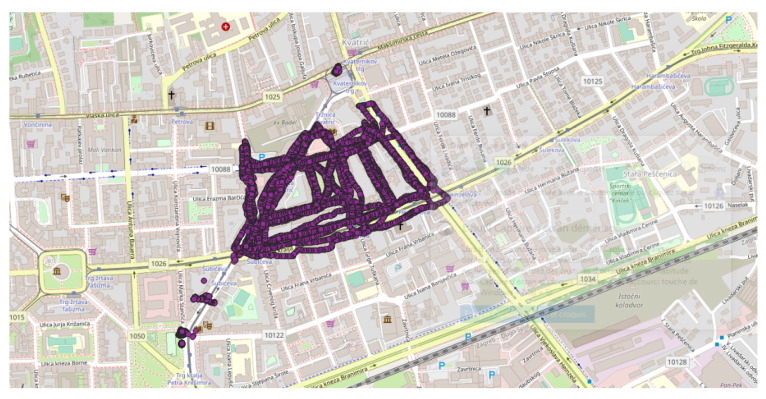
Representation of the cluster found as part of the event (3 tracks/6417 points) in Zagreb (Croatia), in March 2018 [46]; represented area of approximately 1 km × 2 km. All the data are extracted from the NoiseCapture database [41].

Another experiment, involving the comparison of several smartphone noise measurement applications was conducted in 2018 [46]. Measurements were performed over three periods of one day (7:00–9:00, 15:00–17:00 and 19:00–21:00), twice, in an area of the city of Zagreb, Croatia. The reference [46], which is a student report, does not give enough indication about the sampling of the measurements and the exact date of the measurements. Nevertheless, applying our methodology, a cluster was identified, which corresponds to a part of this experimentation (Figure 12). This cluster is located in the proximity of Zagreb train station, and the measurements were collected during 12–18 March 2018.

**Figure 13:**
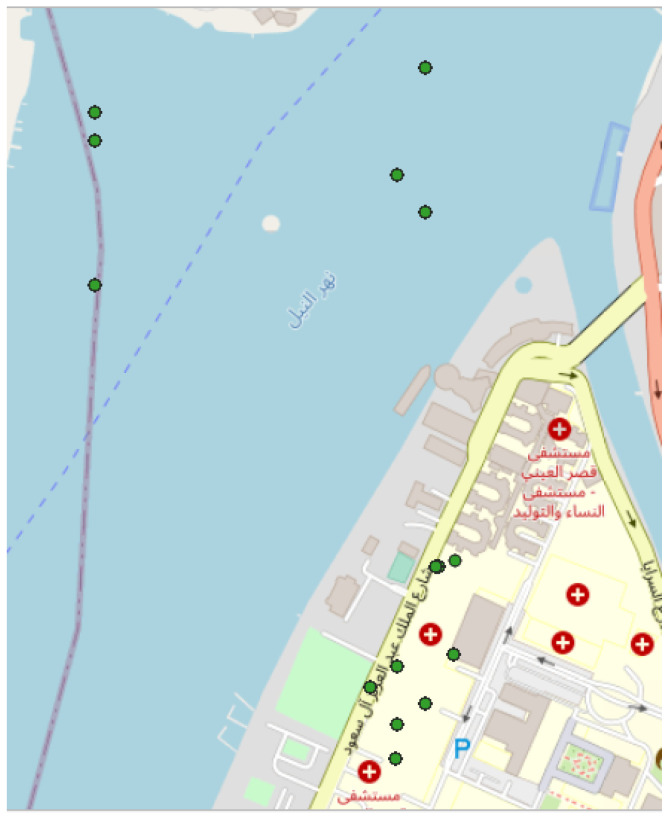
Representation of the cluster found as part of the event (5 tracks, 9418 points) in Cairo (Egypt) in August 2018 [47]. Note that 6309 points are affected by low GPS accuracy due to the measurement inside a building, which makes them poorly positioned on the Nile River; represented area of approximately 950 m × 820 m. All the data are extracted from the NoiseCapture database [41].

The last event that can be found in the scientific literature took place in Cairo, Egypt in August 2018, inside the “Kasr Al Ainy Hospitals” building. These experiments were conducted in order to study the effect of noise pollution on patients undergoing surgery [47]. The experiment appears to have been correctly discovered by the clustering approach, consisting of five tracks with a total of 9418 points and collected by a single user (Figure 13).

## 5. Conclusions

Since 2017, the NC project has collected a large amount of information around the world, which suggests that data may now be useful to evaluate the quality of sound environments. However, the preliminary analysis of the data showed that it is important to quantify the quality of the data in order to control its use [8]. Most of the quality control methods that have been identified in the framework of the NC project for future developments are based on machine learning methods; among them, (semi-)supervised ones seem well-suited. This, however, implies the existence of labelled data to train the models.

Such labelled data can, for example, be obtained during the organization of specific events, called NC parties, for which a supervision of the contributors allows us to ensure that the measurement protocol has been followed and that the smartphones have been calibrated. The data collected during NC parties can thus be considered as reference data and can be used to learn models. However, these reference data are insufficient in number to ensure the quality of learning; additional data are then required in the reference database. The spread of the NC application around the world has also allowed other participants to organize NC party-like events, which would complete the reference database as soon as this data can be identified in the database. In general, the realization of these events generates an increase in the spatial and temporal density of the measurement points, which suggests that clustering-type methods would be well-suited to detect them. Among them, the DBSCAN method was retained in the present paper and tested in several configurations.

The method was first applied in order to verify that the known NC parties could be detected. By judiciously choosing the two main DBSCAN parameters, the search radius of the measurement points (Eps) and the minimum number of points (MinPts), it is thus possible to find 100% of the known NC parties in the form of clusters. In a second step, the method was applied to a selection of countries in order to analyze the typology of the detected clusters. Several events similar to the NC party were indeed detected, but, depending on the value of the processing parameters, a greater number of additional clusters can also be detected without being able to associate them in an obvious way with a particular event. Finally, the method was applied to the whole NC database, with a set of parameters aimed at detecting the most important clusters. More than 2000 clusters were detected in the world, some of which could be associated with events organized in the framework of research published in the literature.

It is clear that the method is very dependent on the parameters Eps and MinPts, and their choice therefore requires the help of an expert, depending on the typology and number of clusters that are expected. It seems, however, possible in the future to modify the DBSCAN method to automatically find the appropriate values for the parameters Eps and MinPts [48,49].

It is also clear that the classical DBSCAN method may return clusters with a lower level of relevance, and other clustering methods would deserve to be compared in terms of performance [50]. One can, for example, cite the ordering points to identify the clustering structure (OPTICS) [51], which sets out to solve one of DBSCAN weaknesses (i.e., detecting meaningful clusters in data of varying density), by performing the clustering after ordering the points in a linear order (e.g., date/time of measurements or spatial directions, for example). The OPTICS method can give better results than DBSCAN, but it increases the computational time due to the initial ordering of data. Instead of focusing on points to build clusters, it could also be interesting to consider trajectories (since a set of measurement points comes from the same track), using line/trajectory spatial clustering methods. Among them, the TRACLUS method [52] would seem to be well adapted in this case since it allows us to group trajectories with common sub-trajectories in the form of clusters. This is usually the case in practice when an NC party is organized locally, since all contributors start collecting data from the same location (from the same street, for example).

Considering the initial objective (i.e., to build a reference database), post-processing may be carried out after the clustering, for example by merging some detected clusters. Indeed, some measurements can belong to the same event but may be grouped into different clusters because they are performed in different places or different periods. This can be solved by investigating the contributors of each cluster to see whether they are the same, or by detecting if the area of the clusters is overlapping (this is the case, for example, when the measurements are collected in a same space but at a different period). In addition, the reference database may be increased by selecting the most important/relevant clusters and then associating the data that are produced independently by the participants to these events. Finally, this would allow the construction of a larger reference database that could be suitable for the use of supervised machine learning methods to develop quality control protocols for the NC data.

## Figures and Tables

**Table 1:** NC parties description.

NoiceCapture Party				All Data		Geo-Localized Data		Not Geo-Localized Data	
**ID**	**NC party Code**	**Contributors**	**Date/Duration**	**Points**	**Tracks**	**Points**	**Tracks**	**Points**	**Tracks**
1	SNDIGITALWEEK	7	20 September 2017	11,523	133	11,192	118	331	15
2	ANQES	4	16–27 October 2017	4479	29	4458	28	21	1
3	FDS2017	2	15 October 2017	1239	6	1209	5	30	1
5	IMS2018	13	28 March 2018	18,793	0	18,758	67	35	0
6	UDC	8	17 April–27 June 2018	6879	56	4509	44	2370	12
9	TEST44	1	2 May 2018	91	3	91	3	0	0
10	UNISA	13	17, 24 May 2018 and 6 June 2018	15,912	149	15,479	141	433	8
11	PNRGM	2	9 June 2018	6089	13	5957	12	132	2
12	AMSOUNDS	2	20, 21 June 2018	693	18	660	17	33	1
13	PNRGM	14	18, 20 July 2018	21,470	100	19,812	92	1658	8
14	FDSSTRAS	5	12, 13 October 2018	2967	31	2909	25	58	6
15	AGGLOBASTIA	19	4 October–26 November 2018	59,838	507	58,771	506	1067	1
17	FDSNTS	7	12–14 October 2018	5916	66	5840	61	76	5
18	H2020	11	6–9 December 2018	22,060	89	19,869	88	2191	1
19	UDC	20	25 February–5 April 2019	5866	138	4946	108	920	30
20	MSA	9	10 January 2019	1885	9	1883	9	2	0
21	GEO2019	43	12–14 March 2019	63,521	420	62,199	409	1322	11
22	IMS2019	23	28, 29 March 2019	17,309	192	14,161	189	148	3
23	FPSLYO	11	6, 17, 18 May 2019	10,285	34	9548	31	737	3
24	SSSOROLL2019	68	16 April–19 May 2019	36,272	372	35,253	361	979	11
26	UNISA	20	24 May 2019	23,220	332	22,937	328	283	4
27	FDSSTRAS	3	12, 13 October 2019	1771	7	1730	6	41	1
28	H2020	9	4–8 December 2019	32,948	39	31,659	35	1289	4
29	UDC	9	3, 5, 6 March 2020	2099	73	2036	71	63	2
30	MSA	10	23 January 2020	3665	10	3659	9	9	1
31	CICAM	–	–	–	–	–	–	–	–
32	UDC_COVID	33	5–20 May 2020	14,785	249	14,691	249	94	0

**Table 2:** Each line corresponds to a simulation with specific DBSCAN parameters (Eps and MinPts) and filtering variables (time window, accuracy, and zone), in order to determine if the clustering analysis is able to detect an NC party. The number and percentage of the detected NC points/tracks in the main cluster and possible secondary clusters are given, as well as the missing NC points/tracks (non-clustered NC data), and the extra points/tracks in the main cluster (i.e., points/tracks that are not linked to the corresponding NC party). Only 22 out of more than 600 results are displayed in this table. Notations: “Time window”: “M” month; “W” week; “D” day; Country: “FR” France; “SP” Spain; “IT” Italy; “NL” The Netherlands.

	NoiceCapture Party			DBSCAN Parameters and Filtering Variables					Main Cluster		Secondary Clusters		Non Clustered Data		Nb Of	Extra Data in the Main Cluster	
**#**	**ID**	**Points**	**Tracks**	**Eps**	**MinPts**	**Time Window**	**Acc.**	**Zone (Country)**	**Points**	**Tracks**	**Points**	**Tracks**	**Points**	**Tracks**	**Clusters**	**Points**	**Tracks**
1	1	10,700	113	50	20	1 M (September 2017)	Off	PdL (FR)	93.5%	91.2%	6.5%	8.8%	0%	0%	7	12,212	76
2	1	10,700	113	50	100	1 M (September 2017)	Off	PdL (FR)	93.5%	91.2%	5.9%	8%	0.6%	0.8%	2	12,212	76
3	1	10,700	113	50	700	1 M (September 2017)	Off	PdL (FR)	93.5%	91.2%	0%	0%	6.5%	8.8%	1	12,212	76
4	1	10,700	113	100	20	1 M (September 2017)	Off	PdL (FR)	97.2%	96.5%	2.8%	3.5%	0%	0%	2	17,147	119
5	1	10,700	113	500	20	1 M (September 2017)	Off	PdL (FR)	99.8%	100%	0.2%	0%	0%	0%	2	17,148	119
6	1	10,700	113	4000	20	1 M (September 2017)	Off	PdL (FR)	100%	100%	0%	0%	0%	0%	1	17,244	122
7	1	9380	89	50	20	1 M (September 2017)	On	PdL (FR)	100%	100%	0%	0%	0%	0%	7	11,828	70
8	1	10,700	113	50	20	1 W (18–24 September 2017)	Off	PdL (FR)	93.5%	91.2%	6.5%	8.8%	0%	0%	7	11,315	74
9	1	9380	89	50	20	1 W (18–24 September 2017)	On	PdL (FR)	100%	100%	0%	0%	0%	0%	1	10,956	68
10	1	10,700	113	3000	20	1 D (20 September 2017)	Off	PdL (FR)	100%	100%	0%	0%	0%	0%	1	16,231	117
11	3	1209	5	3000	20	1 D (15 October 2017)	Off	PdL (FR)	100%	100%	0%	0%	0%	0%	1	19	1
12	5	18,758	67	3000	20	1 D (28 March 2018)	Off	Quimper (FR)	97.3%	100%	2.7%	0%	0%	0%	2	15,044	114
13	6	4509	44	3000	20	3 M (April–June 2018)	Off	Coruña (SP)	99.4%	95.5%	0.6%	4.5%	0%	0%	4	718	35
14	9	91	3	3000	20	1 M (May 2017)	Off	PdL (FR)	100%	100%	0%	0%	0%	0%	1	0	0
15	10	15,479	141	3000	20	2 M (May–June 2018)	Off	Fisciano (IT)	100%	100%	0%	0%	0%	0%	1	9691	77
16	11	5957	12	3000	20	1 D (9 June2018)	Off	Elven (FR)	100%	100%	0%	0%	0%	0%	1	0	0
17	12	660	17	3000	20	1 W (18–24 June 2018)	Off	Amsterdam (NL)	100%	100%	0%	0%	0%	0%	1	495	12
18	13	19,812	92	3000	20	1 W (16–22 July 2018)	Off	Morbihan (FR)	99.8%	100%	0%	0%	0.2%	0%	1	1021	21
19	13	16,374	84	3000	20	1 W (16–22 July 2018)	On	Morbihan (FR)	100%	100%	0%	0%	0%	0%	1	961	21
20	14	2909	25	3000	20	1 W (8–14 October 2018)	Off	Strasbourg (FR)	100%	100%	0%	0%	0%	0%	1	1028	21
21	15	58,771	506	3000	20	2 M (October–November 2018)	Off	Corse (FR)	100%	100%	0%	0%	0%	0%	1	9654	124
22	17	5840	61	3000	20	1 W (8–14 October 2018)	Off	PdL (FR)	100%	100%	0%	0%	0%	0%	1	1669	35
23	24	35,253	361	5000	20	2 M (April–May 2019)	Off	Catalonia (SP)	50.7%	79.9%	48.8%	19.5%	0.5%	0.6%	9	25,578	246

**Table 3:** DBSCAN approach for the data collected in Peru: 8 clusters are found (see also Figure 6).

Cluster Nb	Tracks	Points	Contributors	Month (Year)	Nearest City	Comments
1	10,740	108,785	23	October (2018)	Cajamarca	
2	248	2334	3	November (2018)	Cajamarca	3 users were part of Cluster 1
3	23	3425	2	March (2019)	Lima	
4	1124	1	1	March (2019)	Lima	
5	16	2171	1	May (2019)	Lima	Same users as for Cluster 3
6	1	295	1	May (2019)	Lima	Same users as for Cluster 4
7	20	6957	2	November (2019)	Lima	Same users as for Clusters 3 and 4
8	4	206	1	November (2019)	Lima	Same users as for Cluster 3

**Table 4:** Number of clusters found by the DBSCAN approach in function of the MinPts parameters by filtering the data by month or by week. The number of clusters with one track or one or two contributors is also given, as well as the clusters with a regular daily collection (i.e., between 2 and 5 tracks per day). The number in parenthesis is the number of NC party events in the country. “Failed NC party” means that all the points of the event were not clustered, while “Partly failed NC” means that at least 70% of the event was clustered. When “Failed NC party” and “Partly failed NC” are both equal to zero, the NC parties are fully clustered.

Country	Eps	MinPts	Period	Number of Clusters						
				Total	1 Track	1 Contributor	2 Contributors	Regular Daily Collection	Failed NC party	Partly Failed NC party
France	3000	20	Month	5204	2416	4370	471	635	0 (18)	0 (18)
	3000	200	Month	1852	429	1278	260	143	1 (18)	0 (18)
	3000	5000	Month	224	19	98	28	29	3 (18)	0 (18)
	3000	10,000	Month	125	8	42	13	27	7 (18)	0 (18)
	3000	5000	Week	297	32	164	41	0	4 (18)	1 (18)
	3000	10,000	Week	125	12	61	14	0	8 (18)	1 (18)
Italy	3000	20	Month	564	270	525	27	5	0 (2)	0 (2)
	3000	200	Month	155	34	129	14	4	0 (2)	0 (2)
	3000	5000	Month	16	3	11	1	0	0 (2)	0 (2)
	3000	10,000	Month	9	1	6	0	0	0 (2)	0 (2)
	3000	5000	Week	15	2	10	1	0	0 (2)	0 (2)
	3000	10,000	Week	7	0	4	1	0	0 (2)	0 (2)
United	3000	20	Month	1094	509	1024	51	22	–	–
Kingdom	3000	200	Month	440	122	389	32	24	–	–
	3000	5000	Month	77	19	62	4	5	–	–
	3000	10,000	Month	48	7	37	4	1	–	–
	3000	5000	Week	111	23	99	9	0	–	–
	3000	10,000	Week	57	10	49	6	0	–	–
Peru	3000	20	Month	83	40	72	9	2	–	–
	3000	200	Month	17	3	11	4	0	–	–
	3000	5000	Month	2	0	0	1	0	–	–
	3000	10,000	Month	1	0	0	0	0	–	–
	3000	5000	Week	2	0	0	1	0	–	–
	3000	10,000	Week	1	0	0	0	0	–	–

## Data Availability

The data presented in this study are openly available in the NoiseCapture database extraction from 29 August 2017 until 28 August 2020 (3 years) at https://doi.org/10.25578/J5DG3W.
